# Diffusion Model of a Non-Integer Order *PI^γ^* Controller with TCP/UDP Streams

**DOI:** 10.3390/e23050619

**Published:** 2021-05-16

**Authors:** Dariusz Marek, Adam Domański, Joanna Domańska, Jakub Szyguła, Tadeusz Czachórski, Jerzy Klamka

**Affiliations:** 1 Department of Distributed Systems and Informatic Devices, Faculty of Automatic Control, Electronics and Computer Science, Silesian University of Technology, Akademicka 16, 44-100 Gliwice, Poland; dariusz.marek@polsl.pl (D.M.); adam.domanski@polsl.pl (A.D.); 2 Institute of Theoretical and Applied Informatics, Polish Academy of Sciences, Bałtycka 5, 44-100 Gliwice, Poland; joanna@iitis.pl (J.D.); tadek@iitis.pl (T.C.); jerzy.klamka@iitis.pl (J.K.)

**Keywords:** active queue management, diffusion approximation, fractional controller *PI^γ^*, internet, TCP/IP and UDP

## Abstract

In this article, a way to employ the diffusion approximation to model interplay between TCP and UDP flows is presented. In order to control traffic congestion, an environment of IP routers applying AQM (Active Queue Management) algorithms has been introduced. Furthermore, the impact of the fractional controller $PI^{\gamma }$ and its parameters on the transport protocols is investigated. The controller has been elaborated in accordance with the control theory. The TCP and UDP flows are transmitted simultaneously and are mutually independent. Only the TCP is controlled by the AQM algorithm. Our diffusion model allows a single TCP or UDP flow to start or end at any time, which distinguishes it from those previously described in the literature.

## 1. Introduction

Network protocols control the transfer of information between a transmitter and receiver. They have to fulfil many functionalities, for example, ensure the correctness of transmitted data and increase channel capacity. This article focuses on the problem of transmission efficiency. Traffic intensity has a stochastic nature. The protocols should adapt the transmission to the limited bandwidth and deterministic mechanisms of computer networks. The goal is to minimize information loss due to packet drops in overflowing buffers. These control activities are based on the exchange of information between sender and receiver. The article analyses two approaches to increase the efficiency (by minimizing packet loss) in the wide-area network:

- a mechanism for controlling the speed of sending information at the transmitter level, due to the TCP protocol [1];
- removing data from the network regulated by the AQM mechanism on the IP level [2].

Due to the rapid development of network communication technology, more and more attention has been focused on the problems of congestion control. Congestion is an important factor affecting a network’s Quality of Service (QoS) and reducing its performance. The Transport Control Protocol (TCP) has already been used in Internet applications for more than 30 years. During this time, several congestion control algorithms have been developed to meet the requirements of a constantly changing computer network. Active Queue Management (AQM) is a network approach to congestion prevention that works in combination with the TCP protocol. The most effective congestion control occurs when the AQM mechanism and TCP protocol work together [3].

The earliest of the AQM algorithms is called Random Early Detection (RED) [4]. In the past twenty years, many AQM mechanisms have been proposed. These mechanisms can be classified into three categories [5,6]: heuristic, optimization and control theory approach. The heuristic approach towards the AQM mechanism heavily depends on intuition. Examples of such mechanisms are inter alia, BLUE algorithm [7], hyperbola RED (HRED) [8], and Yellow algorithm [9]. These algorithms aim to improve packet loss, fairness, network utilization, and adaptability to different characteristics of the network traffic. The AQM based on the optimization approach was developed by Frank Kelly. His paper [10] transforms the design of the AQM algorithm to a convex optimization problem. Another AQM algorithm elaborated following the optimization theory was the Random Early Marking (REM) [11]. However, the AQM mechanisms based on the optimization theory are usually complex and hard to tune [6]. In this case, it is also difficult to control the instantaneous queue length of the router [12]. To overcome these effects, researchers have resorted to the control theory. The exact type of a $PI^{\gamma }$ controller used in this article belongs to the family of AQM mechanisms based on this theory, described in Section 2.

The dynamic model of the TCP behavior is required to enable the application of the control theory principles to AQM. The transient analysis should be performed in the model to provide us with time-dependent behavior of flows and queues. Apart from discrete time simulation, which is very time-consuming in the case of transient-state analysis, fluid flow or diffusion approximation methods can be used. Usually, due to its simplicity, the fluid flow method is the one used to model TCP networks [13,14,15,16]. The diffusion approximation, on the other hand, offers more accurate results. In the diffusion approximation model, traffic flows are determined by their mean and variance. On top of that, second-order partial equations describe queue changes.

In this paper, the transient behavior of the AQM mechanisms is analyzed via extending our earlier models presented in [17]. Comparing with previous results, the consideration of independent TCP/UDP streams that can start and end at any time constitutes a novelty. The diffusion approximation method is employed to trace the behavior of the AQM mechanisms applied to control Internet traffic. We are able to describe the features of RED, NLRED, PI and $PI^{\gamma }$ that were not possible to notice in an open-loop or a fluid-flow scenario. Table 1 presents main approaches to AQM based on PI and non-integer order $PI^{\gamma }$ controllers.

This paper is organized as follows: In Section 2 one can find the literature overview of AQM mechanisms based on the control theory approach. This section also describes an embedding of our model in the area of diffusion modelling. Section 3 gives a brief description of the AQM mechanism used in this paper. Section 4 describes our diffusion model of the $PI^{\gamma }$ controller with TCP/UDP streams. In Section 5, numerical results are presented. Section 6 concludes our work.

## 2. Background and Related Work

### 2.1. AQMs Based on the Control Theory Approach

Yes, it should be subsection, thank you. The detection and mitigation of congestion are some of the biggest problems in the computer networks domain [3]. Many mechanisms have been created to solve these issues. They are based on different operating principles. Most of the congestion avoidance mechanisms have been implemented in transport layer protocols such as TCP [26]. UDP applications, such as voice or video traffic, do not have any congestion avoidance mechanisms. In the case of such applications, it is possible to use the open-loop hop-by-hop backpressure strategy. In accordance with this strategy, a congested node broadcasts backpressure messages to upstream nodes to reduce their transmission rate [27]. This method is useful for bandwidth control but, unfortunately, introduces transmission delays. Therefore, there are many attempts to improve these kinds of algorithms, for example, [28]. Back pressure algorithms can be used in wireless sensor networks (WSNs) because of their limited need for: computation resources, storage, energy and communication bandwidth [27,29]. The TCP protocol and the back pressure mechanism are incompatible due to a mismatch between the TCP congestion control mechanism and the back pressure queue size based routing [26]. As a result, wider use of this solution in the Internet would require changes in the TCP protocol [26].

The traffic control in TCP/IP network is, in fact, a closed-loop algorithm in which AQM algorithm plays the role of a controller. The performance and dynamics of network connections can be studied using the control theory to improve their stability and to reduce the reaction time. Several feedback control algorithms have been developed. The article [30] proposes a fluid flow dynamic model of TCP/RED networks by using stochastic differential equations. Based on this dynamic model, several AQM controllers have been proposed using different control approaches. The article [13] proposes a Proportional-Integral (PI) controller on low-frequency dynamics. Authors of the article [31] propose adaptive Proportional (P) and Proportional-Integral (PI) controller and conclude that PI controller can adapt very well to the large fluctuation of the Internet traffic. The article [32] describes a new variant of the RED mechanism called Proportional-Derivative-RED (PD-RED) that performs better than Adaptive RED. Authors of [19] propose the Proportional-Integral-Differential (PID) controller to speed up the responsiveness of the AQM system. Among them, PI controllers are attracting increased attention because of their computation, and implementation simplicity [18]. The article [6] tries to preserve the simplicity of the PI controller by proposing a self-tuning compensated PID controller.

Traditional calculus is based on integer-order differentiation and integration. Differentiation or integration of non-integer order have been used in many mathematical models of dynamic systems. The article [33] claims that many real dynamic systems are better characterized using a fractional dynamic model. Authors indicate that non-integer order controllers provide better performance than the conventional integer order ones. The article [20] presents the first application of the fractional order PI controller to an AQM strategy. The authors focus on the method for determining the parameter regions where the $PI^{\gamma }$ controller ensures a given modulus margin (inverse of the $H_{\infty }$ norm of the sensitivity function). The article [21] describes an evaluation of the fractional-order $PI^{\gamma }$ controller used as an AQM mechanism. The performance of the controller is evaluated using fluid flow approximation (closed-loop control) and simulation (open loop scenario). The article [34] studies the proper selection of the $PI^{\alpha }D^{\beta }$ parameters to show an influence of the proportional, integral and derivative terms on the controller’s dropping function. A simulation model is used in this article. The article [17] proposes a new model of the $PI^{\gamma }$ controller based on a diffusion approximation approach. This model is able to provide more detailed information on transmission delays than the frequently used fluid flow model.

### 2.2. Diffusion Approximation

Diffusion models refer to the changes of flows defined by their mean and variance. They are more accurate than the fluid flow models, where only the mean value is considered. They use the central limit theorem to justify that the number of arrivals and services at a queueing system tends to the normal distribution and a diffusion process may represent the queue length. The solution of the diffusion equation with parameters depending on the analyzed system approximates a queue distribution. This way, the models easily incorporate general distributions of interarrival and service times and the transient queue behavior.

The diffusion approximation has been used to study the performance evaluation of computer systems and networks for many years. The tutorial [35] describes how the diffusion approximation formalism can be applied to the analysis of some traffic control mechanisms in the ATM network. The article [36] presents the diffusion model of wireless network based on the IEEE 802.11 protocol. In the article [37], the first attempt to model the TCP/RED router using diffusion approximation is made. The article [38] applies diffusion approximation to model the influence of a buffer capacity on Quality of Experience in wireless video connections. In our article [17], a new model of TCP NewReno based on the diffusion approximation method is developed. The combined diffusion approximation and simulation model is proposed in [25]. These models allow analyzing the behavior of a single TCP stream. To the best of our knowledge, there is no diffusion model described in the literature which can be used to analyze the independent TCP/UDP streams.

## 3. RED, NLRED and a Non-Integer Order ${\mathrm{PI}}^{\gamma }$ Controllers

The traffic control built-in a TCP/IP protocol is a typical closed-loop one. The flow of packets emitted by a sender is controlled by the loss probability *p* observed in routers and reported to the sender with a certain delay. The losses decrease the traffic, and their lack increases it. In the case of a passive router, the losses occur when the router queue is full.

The RED family algorithms determine the dropping probability even if there is still a place to store packets but the queue increases. They use the weighted moving average avg computed at packets’ arrival; for packet *i*:

$$avg_{i}=\left(1-w\right)avg_{i-1}+wq_{i}$$

where $avg_{i-1}$ is the moving average computed at the arrival of the previous packet, and $q_{i}$ is the queue seen by the packet *i*. The probability of a loss is for small values of avg $p_{RED}=0$. As avg grows, the probability increases linearly between two thresholds $Min_{th}$ and $Max_{th}$, from 0 to $p_{max}$.

(1) $$P_{RED}\left(avg\right)=p_{max}\frac{avg-Min_{th}}{Max_{th}-Min_{th}}$$

Finally, it becomes $p_{RED}=1$ given $avg>Max_{th}$.

Another modification of RED is NLRED (non-linear RED) algorithm [39]. In this mechanism, the linear packet dropping function is replaced by a quadratic function:

(2) $$p=\left\{\begin{array}{cc} 0 & \mathrm{for}\;avg<Min_{th}\\ {\left(\frac{avg-Min_{th}}{Max_{th}-Min_{th}}\right)}^{2}P_{max} & \mathrm{for}\;Min_{th}\leq avg\leq Max_{th}\\ 1 & \mathrm{for}\;avg>Max_{th} \end{array}\right.$$

Paper [40] proposes another approach to a non-linear packet dropping function. This function is based on the third-degree polynomials instead of the well-known quadratic function. This approach allows to choose the optimal packet dropping function:

(3) $$p\left(avg,a_{1},a_{2},p_{max}\right)=\left\{\begin{array}{cc} 0 & \mathrm{for}\;avg<Min_{th}\\ \varphi_{0}\left(avg\right)+a_{1}\varphi_{1}\left(avg\right)+a_{2}\varphi_{2}\left(avg\right) & \mathrm{for}\;Min_{th}\leq avg\leq Max_{th}\\ 1 & \mathrm{for}\;avg>Max_{th} \end{array}\right.$$

where:

(4) $$\varphi_{0}\left(avg\right)=p_{max}\frac{avg-Min_{th}}{Max_{th}-Min_{th}},$$

(5) $$\varphi_{1}\left(avg\right)=\left(avg-Min_{th}\right)\left(Max_{th}-avg\right),$$

(6) $$\varphi_{2}\left(avg\right)={\left(avg-Min_{th}\right)}^{2}\left(Max_{th}-avg\right)$$

Probability *p* can also be determined directly by the controller by comparing the current queue $q_{i}$ and the queue $q_{0}$ we wish to maintain. Their difference, called error in the control theory, is the input signal to the controller, which may be the classical PID one or, as it has been investigated recently, the proportional-integral $PI^{\gamma }$ of fractional, that is, non-integer order. Fractional derivatives and integrals are known since the times of Leibnitz and recently become used in the control of physical processes [41].

In the case of the $PI^{\gamma }$ controller, the loss probability $p_{i}$ of a packet *i* is equal to:

(7) $$p_{i}=max\left\{0,-\left(K_{P}e_{i}+K_{I}\Delta^{\gamma }e_{i}\right)\right\}$$

It depends on the proportional and integral terms $K_{P},K_{I}$, the error $e_{i}=q_{i}-q$, and the order of integration γ. Their impact is further discussed in the articles [21,22,34,42].

The packet drop probability is determined at discrete moments of packet arrivals. There exists only one definition of the non-integer order discrete differ-integral. It [43] is a generalization of the traditional definition of the difference between the integer-order and the non-integer one and is analogous with the generalization employed in the Grünwald-Letnikov (GrLET) formula [44,45].

For a given sequence $f_{0},f_{1},...,f_{j},...,f_{k}$

(8) ▵γfk=∑j=0k(−1)jγjfk−j

where γ∈R is by and large a non-integer fractional order, $f_{k}$ is a differentiated discrete function, and γj is a generalised Newton symbol which definition looks as follows:

(9) γj=1for j=0γ(γ−1)(γ−2)..(γ−j+1)j!for j=1,2,…

Parameters of the non-integer order $PI^{\gamma }$ controller are presented in Table 2.

## 4. Diffusion Approximation of the TCP and UDP Network Streams

In this section, how to model the AQM router supporting TCP/UDP flows using the diffusion approximation is described. The main goal of the analysis presented below is to model the Active Queue Management based on the answer of $PI^{\gamma }$ controller.

The method of diffusion approximation is used in queueing theory (e.g., [46,47,48,49]) when it is hard to determine a queue distribution. The queue length is replaced by the value of diffusion process *X*(*t*). The probability density function (pdf) of the letter, $f(x,\;t;x_{0})$

(10) $$f\left(x,\;t;x_{0}\right)\;dx=P\left[x\leq X\left(t\right)\;<x+dx|X\left(0\right)=x_{0}\right]$$

of X(t), is given by the diffusion equation:

(11) $$\frac{\partial f(x,t;x_{0})}{\partial t}\;=\frac{\alpha }{2}\frac{\partial^{2}f\left(x,t;x_{0}\right)}{\partial x^{2}}-\beta \frac{\partial f(x,t;x_{0})}{\partial x}$$

and helps us to evaluate the queue distribution. It is usually used in the analysis of G/G/1 queueings systems, that is, having general distributions of interarrival and service times or G/G/1/L, where additionaly the queue is limited to L positions. In the latter case the number of packets in router is in the range [0,L] and therefore the diffusion process is limited by barriers in x=0 and x=L, and the diffusion equation is used in the form [47]:

$$\begin{array}{c} \begin{array}{c} \frac{\partial f(x,t;x_{0})}{\partial t}=\frac{\alpha }{2}\frac{\partial^{2}f\left(x,t;x_{0}\right)}{\partial x^{2}}-\beta \frac{\partial f(x,t;x_{0})}{\partial x}\\ +\lambda p_{0}\left(t\right)\delta \left(x-1\right)\;+\lambda p_{L}\left(t\right)\delta \left(x-L+1\right) \end{array} \end{array}$$

(12) $$\begin{array}{c} \begin{array}{c} \frac{dp_{0}\left(t\right)}{dt}=\lim_{x\to 0}\left[\frac{\alpha }{2}\frac{\partial f(x,t;x_{0})}{\partial x}-\beta f\left(x,\;t;x_{0}\right)\right]-\lambda p_{0}\left(t\right)\;,\\ \frac{dp_{L}\left(t\right)}{dt}=\lim_{x\to L}\left[\frac{\alpha }{2}\frac{\partial f(x,t;x_{0})}{\partial x}-\beta f\left(x,\;t;x_{0}\right)\right]-\mu p_{L}\left(t\right)\;. \end{array} \end{array}$$

In the above equations, $[p_{0}\left(t\right)$, $p_{L}\left(t\right)]$ denote probabilities that the process is in either of barriers, λ and μ are the intensities of jumps from barriers; from x=0 to x=1 and from x=L to x=L−1 corresponding to the arrival of a packet to the empty queue or departure of a packet from the full queue. Parameters β and α are chosen as β=λ−μ, $\alpha =\lambda^{3}\sigma_{A}^{2}+\mu^{3}\sigma_{B}^{2}$, where 1/λ, 1/μ are the first moments of interarrival and service time distributions, and $\sigma_{A}^{2}$, $\sigma_{B}^{2}$ are their varainces, more information plesae see Table 3. This way the changes of the diffusion process and of the queue have the same mean and variance. The steady-state solution of Equation (12) is given in [47] and the transient case is considered in [49].

Below, we expand this model to include a number of independent streams. We assume a queue supports *k* input streams. For each stream, packets arrive at intervals which are described by the distribution $A^{\left(k\right)}\left(x\right)$. The service time distribution is equal to $B^{\left(k\right)}\left(x\right)$. The *k* input stream (k=1,...,K) is described by the distribution $A^{\left(k\right)}\left(x\right)$, (with the average value $1/\lambda^{\left(k\right)}$ and variance $\sigma_{A}^{{\left(k\right)}^{2}}$). The service time of the *k* stream has a distribution $B^{\left(k\right)}\left(x\right)$ with the average value $1/\mu^{\left(k\right)}$ and variance $\sigma_{B}^{{\left(k\right)}^{2}}$. The density functions of these distributions are denoted by $a^{\left(k\right)}\left(x\right)$ i $b^{\left(k\right)}\left(x\right)$. We assume input streams of individual classes are independent. The normal distribution of the number of packets of a *k*-th stream coming over a period of time is approximately equal to $\lambda^{\left(k\right)}t$, with a variance satisfying the equation $\lambda^{{\left(k\right)}^{3}}\sigma_{A}^{{\left(k\right)}^{2}}t=\lambda^{\left(k\right)}C_{A}^{{\left(k\right)}^{2}}t$. The number of packets of all streams that arrived during this time has also a normal distribution with the average value $\lambda t=\sum_{k=1}^{K}\lambda^{(k)t}$ and a variance satisfying the equation $\lambda C_{A}^{2}t=\sum_{k=1}^{K}\lambda^{\left(k\right)}C_{A}^{{\left(k\right)}^{2}}t$. So, the parameters of the totality of streams are:

(13) $$\lambda \left(t\right)=\sum_{k=1}^{K}\lambda^{\left(k\right)}\left(t\right),\;\;C_{A}^{2}=\sum_{k=1}^{K}\frac{\lambda^{\left(k\right)}\left(t\right)}{\lambda (t)}C_{A}^{{\left(k\right)}^{2}}$$

where $\frac{\lambda^{\left(k\right)}\left(t\right)}{\lambda (t)}$ is the probability that a given packet belongs to a stream *k* which allows us to determine the resultant service time parameters:

(14) $$\frac{1}{\mu }=\sum_{k=1}^{K}\frac{\lambda^{\left(k\right)}}{\lambda }\frac{1}{\mu^{\left(k\right)}},\;C_{B}^{2}=\mu^{2}\sum_{k=1}^{K}\left[\frac{\lambda^{\left(k\right)}}{\lambda }\frac{1}{\mu^{{\left(k\right)}^{2}}}\left(C_{B}^{{\left(k\right)}^{2}}+1\right)\right]-1,$$

and then the parameters α, β of the diffusion equations:

$$\beta \left(t\right)=\lambda \left(t\right)+\mu ,\;\;\alpha \left(t\right)=\lambda \left(t\right)C_{A}^{2}+\mu C_{B}^{2}$$

The distribution p(n)≈f(n) specifies the number of packets of all streams in the queue, and the probability that there are *v* packets which belong to the *k*-th stream in the queue equals to:

(15) p(k)(v)=∑n=v∞[p(n)nv(λ(k)λ)v(1−λ(k)λ)n−v],

where k=1,...,K.

In the article, the case of two kinds of input streams is presented. UDP stream is a CBR stream with assumed number of packets sent per time unit ($\lambda_{UDP}\left(t\right)$ is constant) and TCP stream for which the input intensity changes according to the TCP NewReno congestion control algorithm ($\lambda_{TCP}\left(t\right)$ is shaped by the AQM mechanism). When the diffusion model is considered, the total intensity of the input stream is equal to the sum of intensities of the components.

In the case of two input streams, one TCP and one UDP, the mean and the variance of input distribution for a queue is calculated as follows:

$$\beta \left(t\right)=\lambda_{TCP}\left(t\right)-\lambda_{UDP}\left(t\right)-\mu$$

$$\begin{array}{c} \begin{array}{c} \alpha \left(t\right)=[\lambda_{TCP}\left(t\right)+\lambda_{UDP}\left(t\right)]\\ {}[\frac{\sigma_{A_{TCP}}^{2}\left(t\right)\lambda_{TCP}^{3}\left(t\right)+\sigma_{A_{UDP}}^{2}\left(t\right)\lambda_{UDP}^{3}\left(t\right)}{\lambda_{TCP}\left(t\right)+\lambda_{UDP}\left(t\right)}]+\mu C_{B}^{2} \end{array} \end{array}$$

(16) $$\alpha \left(t\right)=\sigma_{A_{TCP}}^{2}\left(t\right)\lambda_{TCP}^{3}\left(t\right)+\sigma_{A_{UDP}}^{2}\left(t\right)\lambda_{UDP}^{3}\left(t\right)+\mu C_{B}^{2}$$

The TCP NewReno/AQM model based on the diffusion approximation works as follows: The diffusion approximation gives the distribution of the router’s queue at time *t*. The mean value of this queue length modifies the packet rejection probability. This probability affects the intensity l of the input stream because the congestion window increases by one for lossless transmission or halves for packet loss. We assume that the flow λ, the size of the congestion window *W*, and the delay q/μ of the router are linked together in the following way:

(17) $$\lambda =\frac{W\mu }{q}$$

where 1/μ is the mean service time. In the diffusion model, the calculations are carried out inside the intervals of length Δt=1/λ. When λ changes, the length of the interval changes as well. We obtain a queue distribution at time $t_{i}$ at the end of the interval *i*. Based on it, we get the average queue length $E[q_{i}]$ and then the probability of packet rejection $p_{i}$ which defines new value of λ: $\lambda_{i+1}=\lambda_{i}+\Delta \lambda_{i}$ due to the AQM algorithm, where

(18) $$\Delta \lambda_{i}=\frac{\mu_{i}}{E[q_{i}]}-\frac{\lambda_{i}^{2}}{2}\frac{E[q_{i}]}{\mu }p_{i}.$$

**Figure 1:**
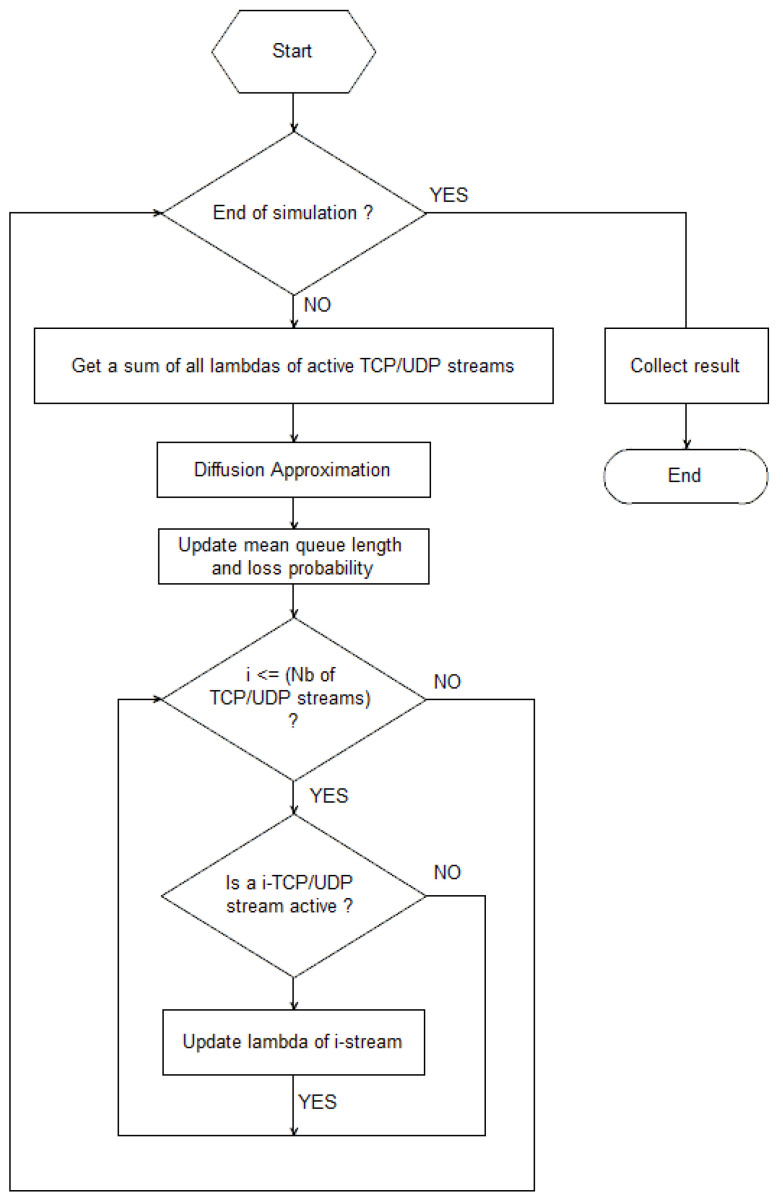
Flowchart of our model behavior.

Over the time $t_{i+1}=t_{i}+1/\lambda_{i}$, we repeat the calculations for a new value of λ. In the model presented in this article it is assumed that the *i*-th flow can start or end a transmission at any moment. The change in the source intensity Δλ in the time *t* affects the dispatching time and the queue length. The algorithm of these calculations is presented in Figure 1.

## 5. Numerical Results

In this chapter, the results obtained through the method described above are discussed. We employ the proposed numerical scheme to obtain the transient behavior of TCP window dynamics and queue length. The results for various types of input streams and AQM mechanisms (RED, NLRED, PI, $PI^{\gamma }$) are discussed.

We assume the following parameters of the AQM buffer: $Min_{th}=10$, $Max_{th}=20$, buffer size (measured in packets) =30.

The parameters of RED are as follows: weight parameter w=0.008, $p_{max}=0.02$. Article [50] shows the impact of RED parameters on the network traffic. Using higher values of these parameters results in the increase in network traffic fluctuations. This choice of RED parameters shows more clearly the influence of TCP/UDP flows on the AQM queue behavior [17]. These parameters are slightly different than those proposed in the literature [51]. The NLRED parameters are as follows: $a_{1}=0.00042$, $a_{2}=-0.0000038$ and $p_{max}=0.855$. Article [40] proposes using parameters with such values to achieve the best transmission performance.

The parameters of $PI^{\gamma }$: setpoint=10, P=0.0001, I=0.005, γ=−0.4. For γ=−1.0 the PI controller is considered (Table 4). The proper choice of AQM/PI controller parameters is difficult. It strongly affects the packet dropping function (i.e., integral order γ accelerates and strengthens the controller’s response). Proper selection of AQM parameters should help achieve two goals: obtaining desired queue behavior and adaptation to network transmission conditions. The influence of the controller’s parameters on queue behavior is discussed in papers [21,22].

**Figure 2:**
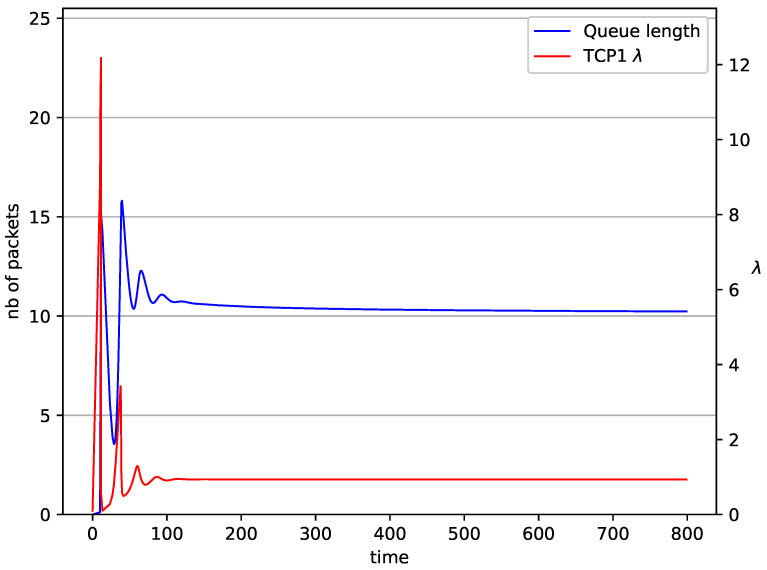
1 TCP with $PI^{\gamma }$ controller.

**Figure 3:**
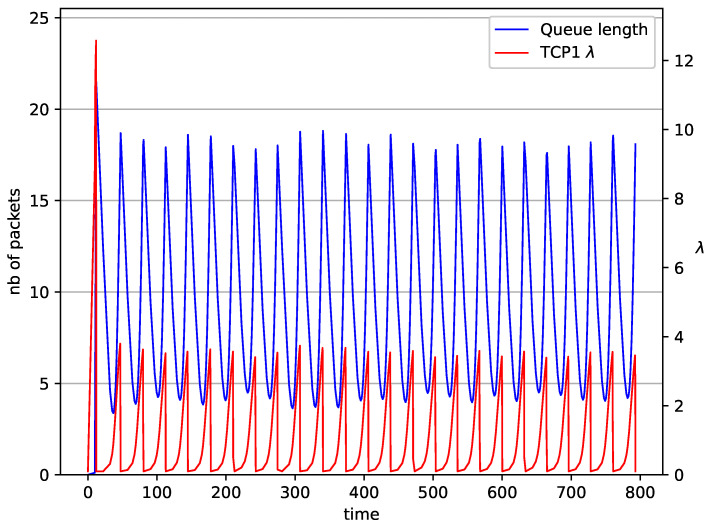
1 TCP with PI controller.

**Figure 4:**
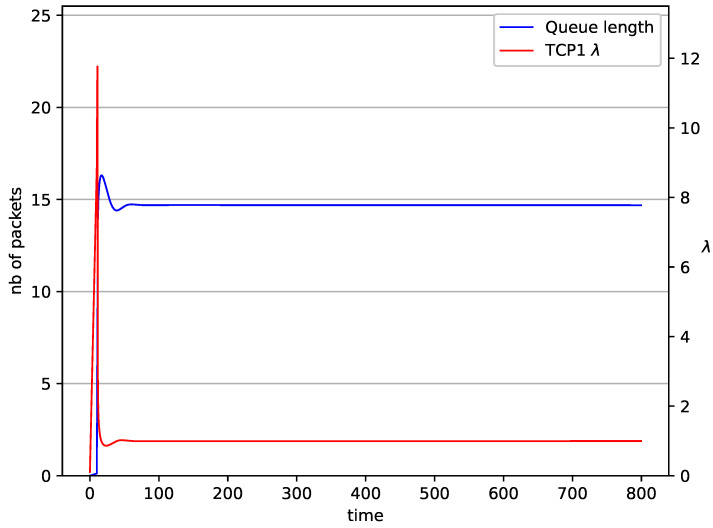
1 TCP with RED controller.

**Figure 5:**
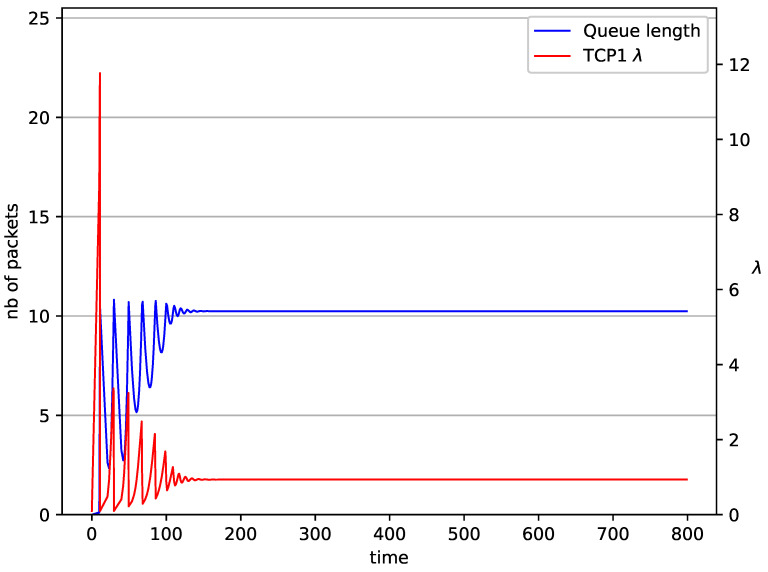
1 TCP with NLRED controller.

**Figure 6:**
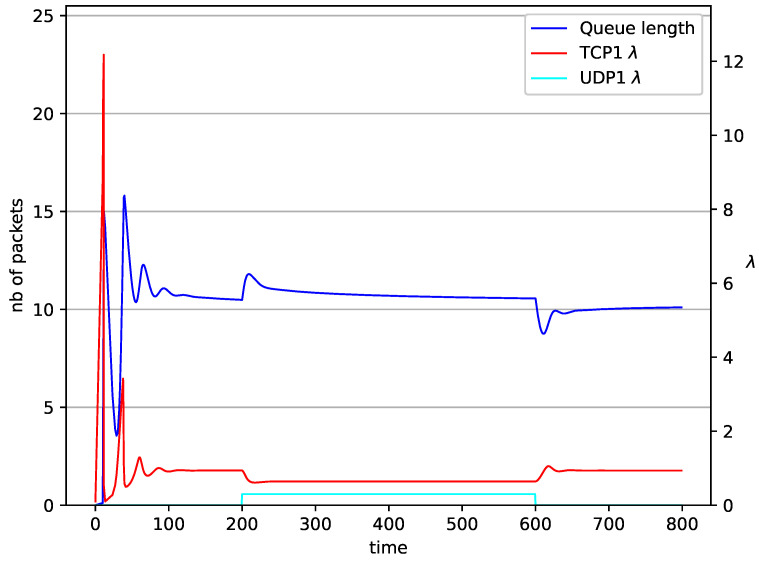
1 TCP and 1 UDP with $PI^{\gamma }$ controller.

**Figure 7:**
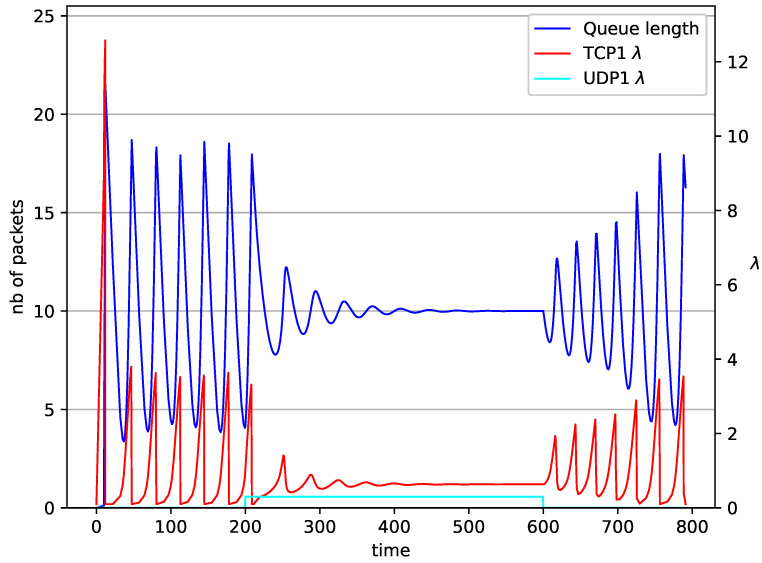
1 TCP and 1 UDP with PI controller.

**Figure 8:**
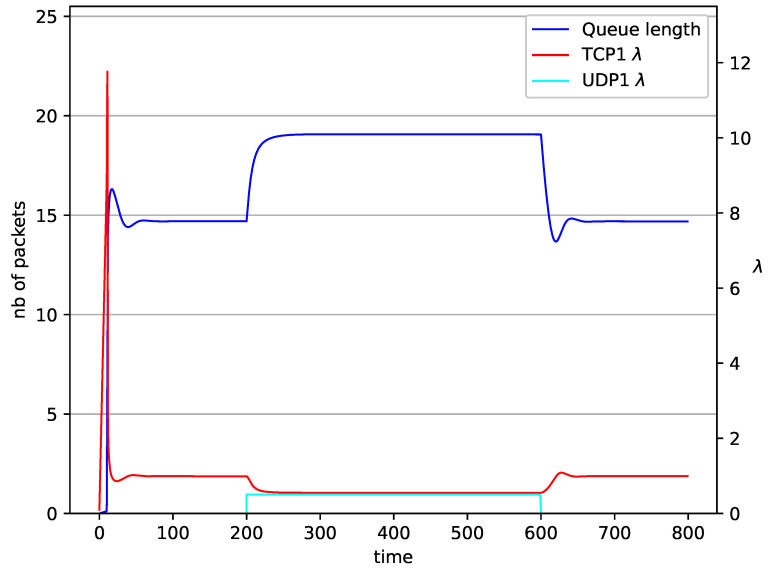
1 TCP and 1 UDP with RED controller.

**Figure 9:**
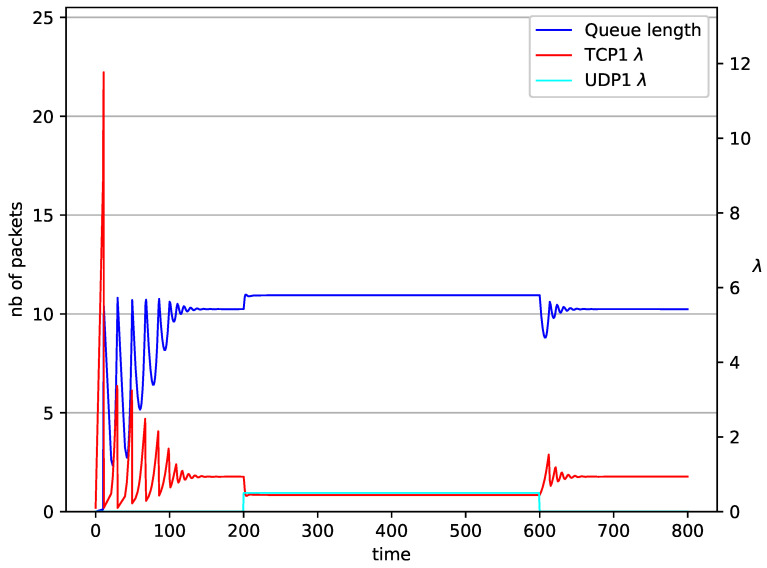
1 TCP and 1 UDP with NLRED controller.

**Figure 10:**
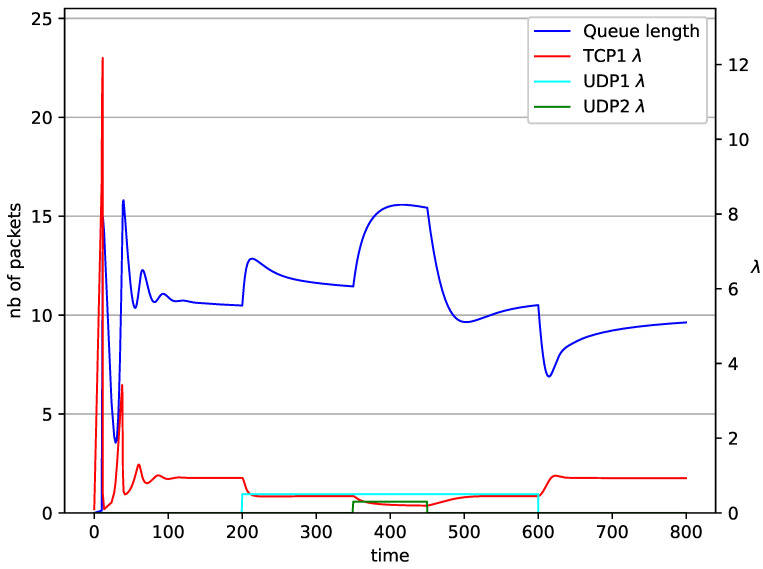
1 TCP and 2 UDP with $PI^{\gamma }$ controller.

**Figure 11:**
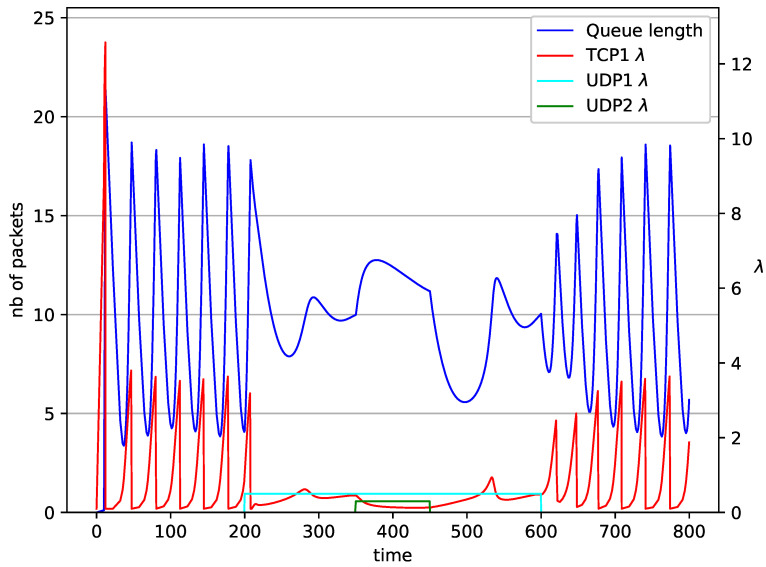
1 TCP and 2 UDP with PI controller.

**Figure 12:**
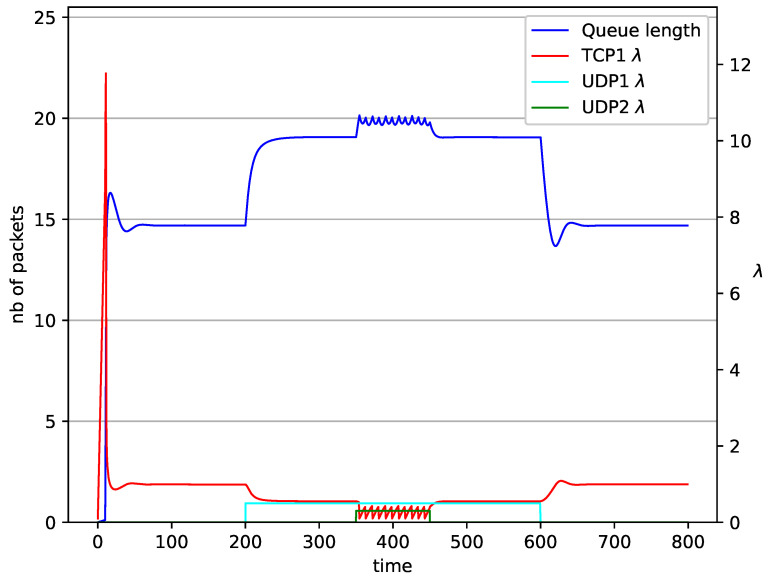
1 TCP and 2 UDP with RED controller.

**Figure 13:**
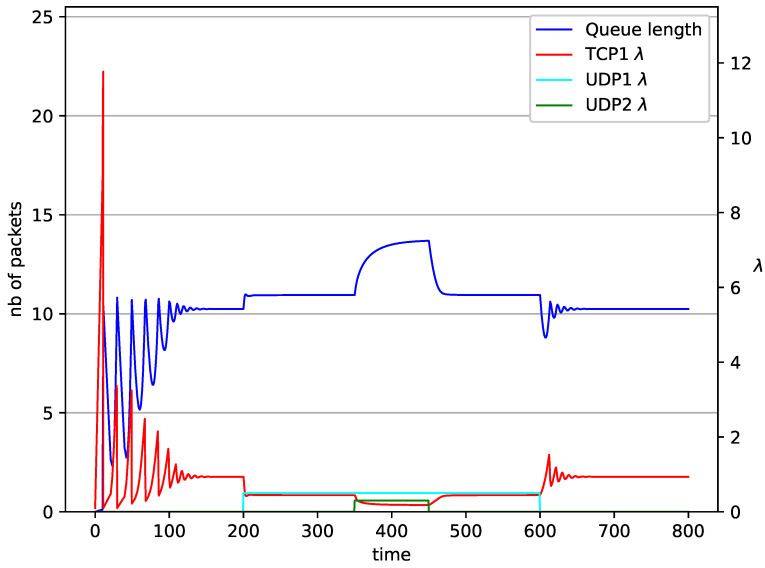
1 TCP and 2 UDP with NLRED controller.

**Figure 14:**
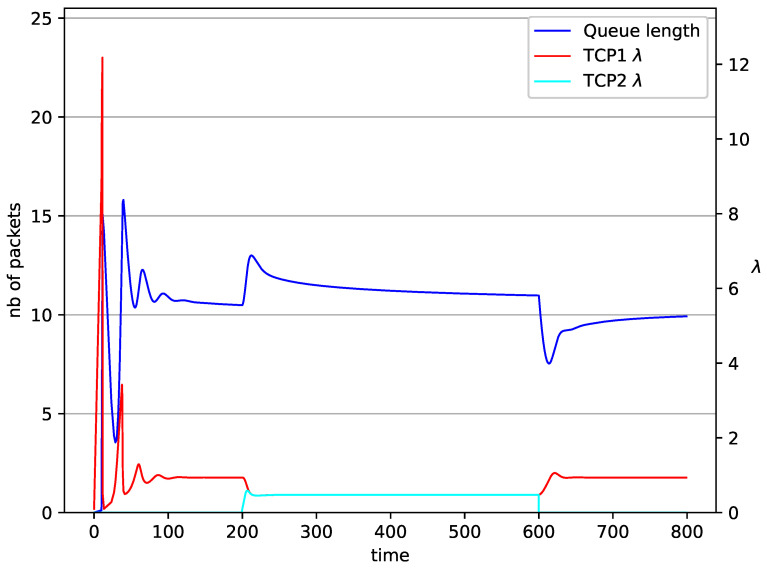
2 TCP with $PI^{\gamma }$ controller.

**Figure 15:**
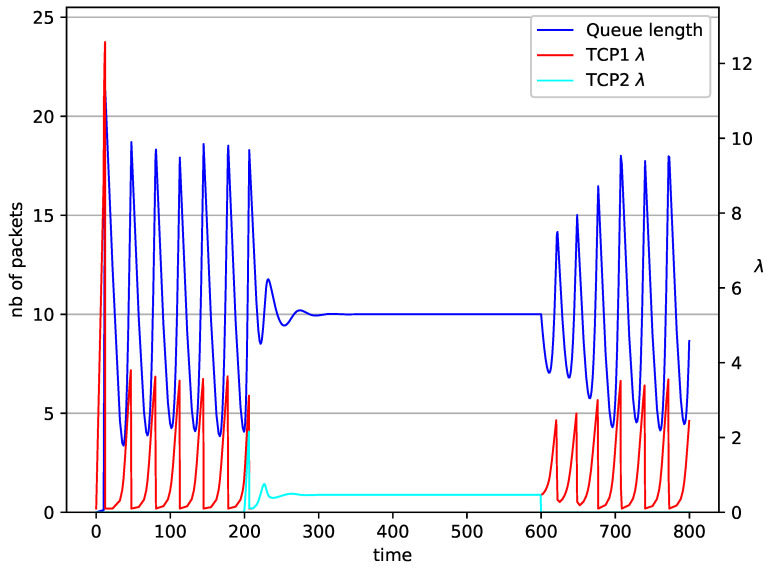
2 TCP with PI controller.

**Figure 16:**
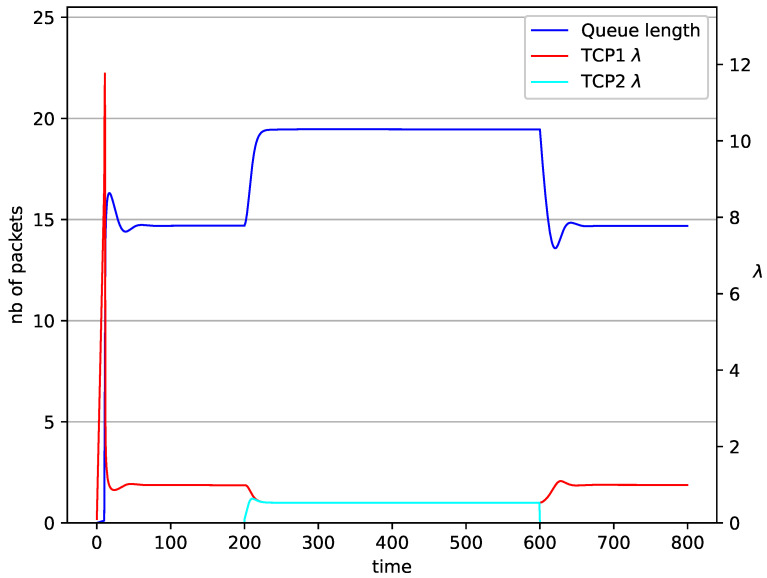
2 TCP with RED controller.

**Figure 17:**
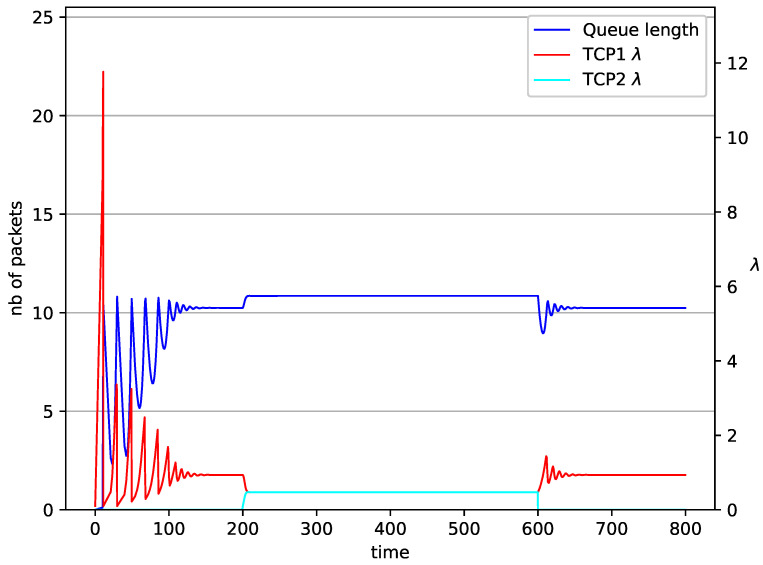
2 TCP with NLRED controller.

Figure 2, Figure 3, Figure 4, Figure 5, Figure 6, Figure 7, Figure 8, Figure 9, Figure 10, Figure 11, Figure 12, Figure 13, Figure 14, Figure 15, Figure 16 and Figure 17 show the evolution of sources intensity and queue length. In Figure 2 the queue behavior in the case of one TCP flow and $PI^{\gamma }$ controller is shown. The source intensity increases until the queue reaches the desired length. Exceeding the desired queue length increases the probability of a packet loss in AQM and thus causes reduction in the source intensity. Consequently, the queue occupancy and packet loss probability decreases. So, the source intensity increases after some time. Source intensity and queue length oscillations continue until a stable state is reached. In the stable state, the average queue length reaches 10.2 packets, and the source intensity reaches λ=1. In the case of a PI controller (Figure 2) the average queue length reaches almost the same value (10.01 packets). Nevertheless, the queue occupancy still oscillates between extreme values. Such oscillations, associated with a big fractional order of the controller, are described in the article [24].

The behaviors of a single TCP stream in cooperation with the RED algorithm (Figure 4) and NLRED (Figure 5) are shown. It is identical to the previous one (Figure 2). The average queue length reaches 14.7 for RED and 10.5 packets for NLRED, the source intensity reaches λ=1. This value is roughly halfway between $Min_{th}$ and $Max_{th}$.

In Figure 6, Figure 7, Figure 8, Figure 9, Figure 10, Figure 11, Figure 12, Figure 13, Figure 14, Figure 15, Figure 16 and Figure 17 the behavior of the TCP/UDP AQM system for several co-transmitted data streams is portrayed.

In Figure 6, Figure 7, Figure 8 and Figure 9 simultaneous transmission of TCP and UDP streams is presented. Figure 6 shows the situation where a queue is controlled by the $PI^{\gamma }$ mechanism. The TCP transmitter starts a transmission at time t=0. The TCP window evolution proceeds, as shown in Figure 2. At t=200 the UDP stream (constant intensity λ=0.5) starts its transmission. Thereby, the intensity of the TCP stream is reduced and the queue occupancy is increased. Over time, the controller attempts to stabilize the queue at the desired level. At t=650, the UDP stream ends its transmission. As a result, the queue length decreases. The end of the UDP transmission also causes the TCP window to grow. Consequently, the queue length increases up to the desired size. When it comes to the PI controller (Figure 7), queue fluctuations occur as previously. They disappear when an additional UDP stream is loaded into the system and reappear once it has finished the transmission. This behavior is also visible in the next experiments. A similar layout of input streams is presented in Figure 8 and Figure 9. In the case of the RED and NLRED algorithms, the commencement of the UDP stream increases the queue length. For the RED mechanism, it reaches the value near $Max_{th}$.

In Figure 10, Figure 11, Figure 12 and Figure 13 the case of one TCP and two UDP streams employed is depicted. TCP works all the time. The first constant bit rate UDP stream (λ=0.5) starts transmission at time t=200 and finishes at t=600. The second UDP stream (λ=0.3) transmits between t=350 and t=450. The second UDP stream causes queue overload. In both cases ($PI^{\gamma }$ and RED queue), the queue’s occupancy increases dramatically. The $PI^{\gamma }$ mechanism slowly stabilizes the queue, reducing the queue size (Figure 11). The RED (Figure 12) and NLRED (Figure 13) mechanisms cannot handle such amounts of input data. In the case of a RED algorithm, the size of the queue oscillates around the point $Max_{th}$.

In Figure 14, Figure 15, Figure 16 and Figure 17 the situation when two TCP streams cooperate is shown. At the moment when the second stream is switched on, the intensity of both sources stabilises at the same level (λ=0.5). In the case of the RED algorithm, the queues occupancies are set at values close to the $Max_{th}=19.45$. For NLRED mechanism, the average queue size reaches 10.1. In the case of the $PI^{\gamma }$ controller, the queue length, after a temporary increase, sags to the desired value. This trend ends when the second source ends its transmission.

## 6. Conclusions

This article describes the use of the diffusion approximation to estimate the behavior of the AQM mechanisms, their influence on the evolution of the TCP congestion window and potential cooperation of more TCP/UDP streams. Analytical methods for assessing network mechanisms known from the literature, with the exception of the fluid flow method, are not very suitable for evaluating TCP mechanisms. In the article [17], we have presented a new approach of using the diffusion approximation to model the behavior of the TCP stream. In this article, we expand the given mechanism with the possibility of evaluation of cooperation of more TCP/UDP streams.

The experiments also show the good points of the fractional order controller $PI^{\gamma }$. Decreasing the integration order of the controller reduces the queue fluctuation.

On the other hand, the new approach gets a completely different view on the cooperation of the TCP/UDP protocol with the AQM mechanisms. Our model allows starting and ending the TCP/UDP transmission at any time. The described approach allows us to observe the dynamics of a transmission when different sources start or end their transmission.

The impact of the AQM algorithm on the dynamics of the TCP window is also presented. Our study shows differences in queue behavior in the case of different AQM mechanisms.

## Figures and Tables

**Table 1:** Main approaches of AQM based on PI and non-integer order $PI^{\gamma }$ controllers.

PI (Simulation) [18]	Study of the TCP/AQM mechanisms based on PI controllers
PID (Simulation) [19]	Evaluation of the AQM based on non-integer order PID controller
$PI^{\gamma }$ (Fluid-Flow) [20]	First application of non-integer order $PI^{\gamma }$ controller to an AQM strategy
$PI^{\gamma }$ (Fluid-Flow/Simulation) [21]	Fluid flow approximation and discrete-event simulation to investigate the influence of the AQM policy based on non-integer order $PI^{\gamma }$ controller on the packet loss probability, the queue length and its variability
$PI^{\gamma }D^{\omega }$ (Simulation) [22]	Model of AQM mechanism based on non-integer order $PI^{\gamma }D^{\omega }$ controller
$PI^{\gamma }$ (Simulation) [23]	Finding optimal parameters of the non-integer order $PI^{\gamma }$ controller used as AQM mechanisms. The optimization was made by using the well-known Hooke and Jeeves direct search method applied for minimization of a multivariate score function
Adapted $PI^{\gamma }$ (Simulation) [24]	Choice of non-integer order $PI^{\gamma }$ controller parameters based on machine learning algorithms. The controller parameters automatically adjust to network traffic parameters (traffic intensity and self-similarity)
TCP $PI^{\gamma }$ (Diffusion) [17]	The diffusion approximation model of the simple TCP traffic. Evaluation (in close loop scenario) of the effectiveness of active queue management (AQM) mechanisms based non-integer order $PI^{\gamma }$ controller
TCP $PI^{\gamma }$ (Combined Diffusion and Simulation) [25]	Combined diffusion approximation and simulation model based on non-integer order $PI^{\gamma }$ controller

**Table 2:** Parameters of the non-integer order $PI^{\gamma }$ controller.

$K_{P}$	Proportional term
$K_{I}$	Integral term
γ	Integral order
$e_{i}$	Error in current slot
$q_{i}$	Actual queue length
*q*	Desired queue length

**Table 3:** Main notations and parameters of the diffusion model.

λ	Intensity of the input traffic
μ	Intensity of packet processing and dispatching
$\sigma_{A}^{2}$	Variance of interarrival time distribution
$\sigma_{B}^{2}$	Variance of service time distribution
$C_{A}^{2}$	Squared coefficient of variation of interarrival time distribution
$C_{B}^{2}$	Squared coefficient of variation of service time distribution
X(t)	Diffusion process
β	Diffusion parameter; βdt is the mean value of changes of X(t) during dt
α	Diffusion parameter; αdt is the variance of changes of X(t) during dt
$f(x,t,x_{0})$	Probability density that the process will be in state *x* at time *t*, for initial conditions $x_{0}$

**Table 4:** $PI^{\gamma }$ and PI controllers coefficients.

	$K_{p}$	$K_{i}$	γ	Setpoint	Type of Controller
1	0.0001	0.005	−0.4	10	non integer order controller
2	0.0001	0.005	−1.0	10	classical controller

## Data Availability

Not applicable.
